# Intermittent torsion of accessory hepatic lobe: An unusual cause of recurrent right upper quadrant pain

**DOI:** 10.4103/0971-3026.63046

**Published:** 2010-05

**Authors:** Kedar Jambhekar, Tarun Pandey, Chhavi Kaushik, Hemendra R Shah

**Affiliations:** Department of Radiology, University of Arkansas for Medical Sciences, Little Rock, AR 72205, USA

**Keywords:** Accessory hepatic lobe, CT scan, liver, magnetic resonance imaging, torsion

## Abstract

An accessory lobe of the liver is a rare congenital anomaly that can undergo torsion and present as an acute surgical emergency. It is rarely diagnosed preoperatively. We report the preoperative utility of CT scan and MRI in the diagnosis and surgical planning of a case of intermittent accessory hepatic lobe torsion.

## Introduction

Accessory hepatic lobe is an extremely rare anomaly.[1] Most cases with accessory liver tissue are not detected since they do not cause symptoms. However, they can give rise to various clinical symptoms like recurrent abdominal pain and impaired liver function.[1] Usually, the diagnosis is made after laparotomy by histopathological confirmation. This case report highlights the role of CT scan and MRI in the diagnosis and pre-operative assessment of this entity.

## Case Report

A 24-year-old, white woman, presented to the emergency room with a 1-day history of intense and constant cramping upper quadrant abdominal pain, nausea, and vomiting.

She had a prior history of several similar episodes of abdominal pain for which no cause had been found. The past surgical history was significant for treatment of malrotation, anterior abdominal wall repair, and removal of a Meckel's diverticulum and appendix at age 11.

At admission, her vital signs and laboratory studies were normal. Her abdomen was soft, nondistended, without rigidity, but she was tender to palpation in the right upper quadrant and epigastric region.

**Figure 1 (A-D):**
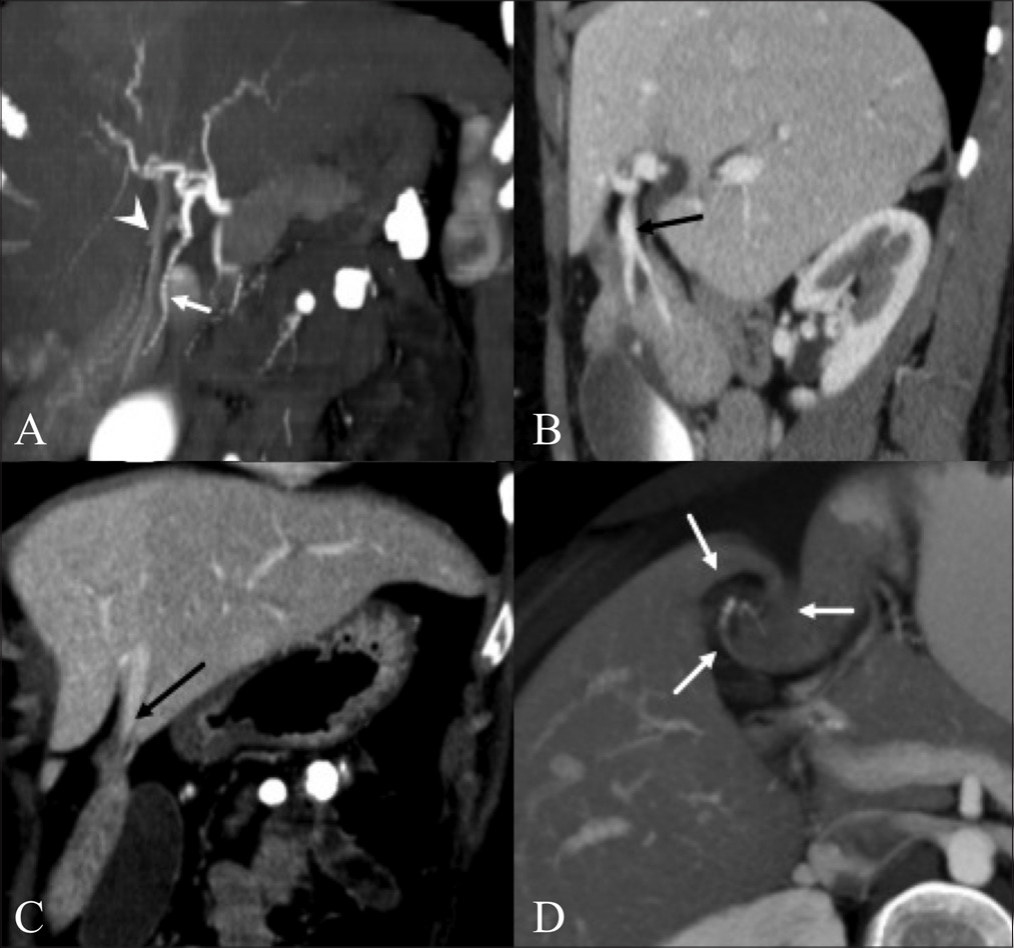
Coronal maximum intensity projection (MIP) arterial-phase CT scan (A) shows anomalous arterial supply (arrow) to the twisted liver segment. The portal venous branches to the accessory lobe are also seen (arrowheads). Sagittal CT scan during the portal venous phase (B) shows an anomalous portal vein branch to the accessory lobe (arrow). An anterior coronal CT scan through the liver (C) demonstrates a branch from the hepatic vein draining the accessory lobe (arrow). Axial thick-slab maximum intensity projection image through the liver (D) demonstrates the twisting of the accessory lobe pedicle (arrows)

**Figure 2 (A,B):**
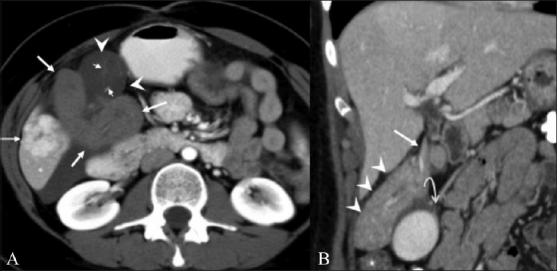
Axial contrast-enhanced CT scan (A) shows torsion of the accessory lobe (arrows), with reduced enhancement compared to the surrounding liver. The gallbladder (GB) appears adherent to the twisted lobe, is anteriorly rotated (arrowheads), and shows wall edema and faint mucosa (small arrows). Focal nodular hyperplasia is incidentally noted (curved arrow). Coronal, reformatted CT scan (B) shows the thin pedicle (arrow) of the accessory liver lobe (arrowheads). Notice the decrease in the GB wall edema (curved arrow) and improved enhancement of the accessory lobe, indicative of interval detorsion

**Figure 3:**
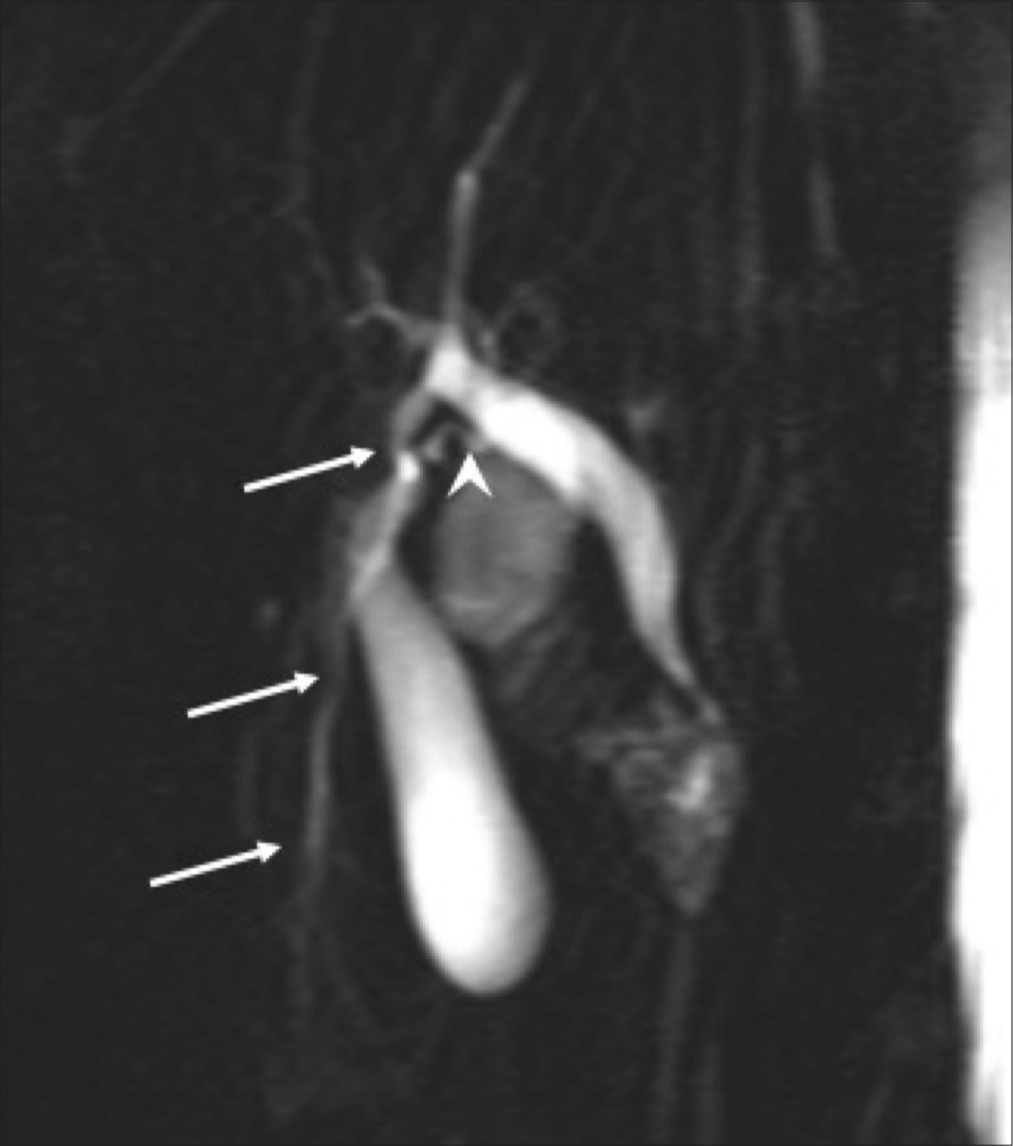
MRI cholangiopancreatography image shows the accessory duct draining into the common hepatic duct (arrows). Notice also a separate, tortuous, cystic duct opening into the bile duct (arrowhead).

A CT scan at an outside facility reported a “right upper quadrant mass.” Since the images were not immediately available, a biphasic contrast enhanced CT scan of the abdomen was performed at our institution. Post-processed, multiplanar reconstructions (MPR) and maximum intensity projection (MIP) thick slab images were also obtained. The CT scan showed a region of liver parenchyma separate from the normal liver and of lower attenuation, attached to the left lobe by a thin stalk of liver tissue that contained a vascular pedicle consisting of hepatic arterial supply from the left main hepatic artery, portal venous supply, hepatic venous drainage directly to the IVC and independent biliary ducts [Figure 1 A–C]. The vascular pedicle was twisted upon itself raising the suspicion of torsion of an accessory lobe of the liver [Figure 1D]. On retrospective comparison with the outside CT scan, the attenuation of the accessory lobe had visually improved [Figure 2A,B]. The hepatic vein was not visualized at the level of the stalk, reflecting vascular compromise. The gallbladder appeared to be adherent to the accessory lobe, with surrounding edema. MRI with magnetic resonance cholangiopancreatography (MRCP) was performed at the surgeon's request to further evaluate and define the anatomy. MRI confirmed the presence of an accessory lobe of the liver and also showed separate arterial and venous anatomy. On MRCP, a separate bile duct was seen draining the accessory lobe into the common hepatic duct [Figure 3].

**Figure 4:**
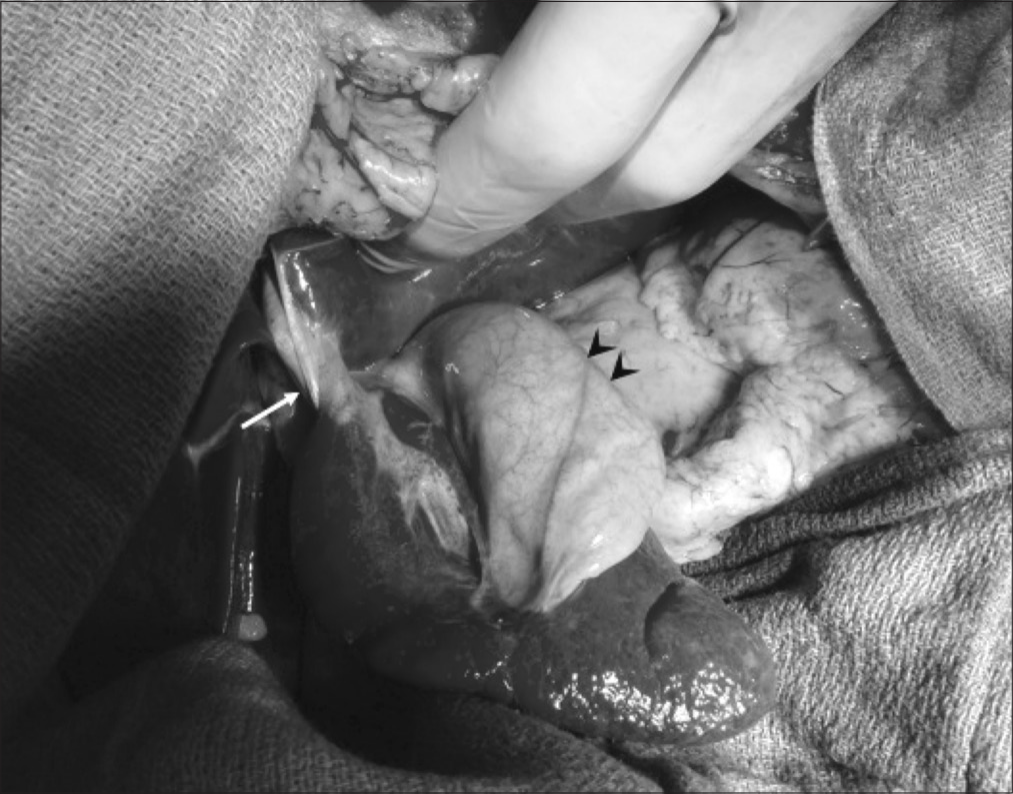
Peri-operative photograph shows the accessory lobe with its pedicle (arrow) and the adherent gallbladder (arrowheads).

During the course of her hospital stay, the patient showed significant improvement in her symptoms and was discharged in a stable condition with a diagnosis of intermittent torsion of an accessory lobe. Elective exploratory laparotomy performed 4 months later confirmed the presence of an accessory hepatic lobe [Figure 4]. The histology revealed normal mature liver tissue. No hepatic infarction or necrosis was identified. The patient has been asymptomatic since surgery.

## Discussion

An accessory hepatic lobe is a rare congenital developmental anomaly that is usually asymptomatic and is seen as an incidental finding at laparotomy.[1,2] There are only a few reported cases of a symptomatic accessory hepatic lobe. Most of these are diagnosed at surgery in patients presenting with nonspecific complaints of recurrent abdominal pain and impaired liver functions.[1,2]

The presence of an accessory hepatic lobe occurs from an error in the formation of the endodermal caudal foregut in the third gestational week and segmentation of the hepatic bud.[3] Anterior abdominal wall defects are frequently associated with accessory hepatic lobes and thought to be secondary to prevention of fusion of the anterior abdominal wall elements by the accessory lobe.[4] There is an increased incidence of prior surgery for repair of omphalocele or gastroschisis.[2,5,6] Our patient had a history of malrotation and repair of the anterior abdominal wall as a child.

An accessory liver is adjacent and attached to the liver by its own mesentery, while an ectopic liver is one that is completely detached from the normal liver parenchyma. While a definite connective tissue stalk attaching the lobe to the rest of the liver was present in our case, a mesenteric covering was not found at surgery. Older literature has described four types of accessory liver: big accessory hepatic lobe (> 30 g), small accessory hepatic lobe (< 30 g), ectopic lobe with no liver connection, and microscopic accessory lobe in the gall bladder wall.[1]

Classification can also be based on the biliary drainage and the presence or absence of a common capsule:

- *Type I*. The separate accessory lobe duct drains into an intrahepatic bile duct of the normal liver.
- *Type II*. The separate accessory lobe duct drains into an extrahepatic bile duct of the normal liver.
- *Type III*. The accessory lobe and the normal liver have a common capsule; the bile duct of the accessory lobe drains into an extrahepatic duct.[5]

Symptomatic accessory hepatic lobe has been reported in infants as young as 23 days of age as well as in patients presenting late in the third decade of life commonly from torsion.[1,6] The usual presentation is an acute abdomen, with right upper quadrant pain with or without a palpable mass. Common differential diagnoses are acute cholecystitis, pancreatitis, duodenal hematoma, intussusception, perforated peptic ulcer, ovarian torsion, wandering spleen, retrocecal appendicitis,[7] and an intra-abdominal tumor.[1] Umbilical hernia and bile duct cysts have been the other reported associations.[3,7]

Accessory hepatic lobe may present as an undetermined soft tissue mass on imaging. Also, torsion complicated by congestion or infarction can further confuse the appearance.[2,7,8] The diagnosis is made after careful evaluation for all hepatic connections on the axial and reconstructed (MIP/ MPR) images. The presence of independent biliary drainage as well as arterial, venous, and portal branches should arouse suspicion of this rare anomaly. As in our case, acute torsion presents on CT scans with decreased or absent enhancement on contrast-enhanced images. Chronic torsion may be associated with cystic changes and congestion with hypertrophy of the accessory lobes.[2,5]Timely surgical intervention is warranted when an accessory hepatic lobe undergoes torsion. Laparotomy can serve as the mainstay of diagnosis as well as treatment. In the majority of cases reported in literature, the gall bladder is usually seen embedded in the accessory lobe and undergoes torsion along with the accessory lobe; in our case, however, the gall bladder was adherent to the accessory lobe probably as a result of multiple previous episodes of inflammation.[7]

In conclusion, it is important for the radiologist to be aware of this entity as a cause of recurrent right upper quadrant acute abdominal pain. Though rare, routine inspection of vascular and biliary drainage of the liver as part of the check list while evaluating all CT abdomen exams will alert the radiologist to this diagnosis when a mass is present in the right upper quadrant. In the correct setting, a past history of an abdominal wall abnormality is also suggestive of the presence of an accessory hepatic lobe. Preoperative diagnosis of this abnormality and mapping its vascular and biliary anatomy help reduce the overall morbidity associated with abdominal surgery.
